# Heritability of decisions and outcomes of public goods games

**DOI:** 10.3389/fpsyg.2015.00373

**Published:** 2015-04-22

**Authors:** Kai Hiraishi, Chizuru Shikishima, Shinji Yamagata, Juko Ando

**Affiliations:** ^1^Faculty of Psychology, Yasuda Women’s UniversityHiroshima, Japan; ^2^Faculty of Liberal Arts, Teikyo UniversityTokyo, Japan; ^3^Faculty of Arts and Science, Kyushu UniversityFukuoka, Japan; ^4^Faculty of Letters, Keio UniversityTokyo, Japan

**Keywords:** public goods game, heritability, twin study, individual differences, behavior genetics, cooperation

## Abstract

Prosociality is one of the most distinctive features of human beings but there are individual differences in cooperative behavior. Employing the twin method, we examined the heritability of cooperativeness and its outcomes on public goods games using a strategy method. In two experiments (Study 1 and Study 2), twin participants were asked to indicate (1) how much they would contribute to a group when they did not know how much the other group members were contributing, and (2) how much they would contribute if they knew the contributions of others. Overall, the heritability estimates were relatively small for each type of decision, but heritability was greater when participants knew that the others had made larger contributions. Using registered decisions in Study 2, we conducted seven Monte Carlo simulations to examine genetic and environmental influences on the expected game payoffs. For the simulated one-shot game, the heritability estimates were small, comparable to those of game decisions. For the simulated iterated games, we found that the genetic influences first decreased, then increased as the numbers of iterations grew. The implication for the evolution of individual differences in prosociality is discussed.

## Introduction

Prosociality is one of the most distinctive features of human beings. We behave cooperatively, even altruistically, toward our conspecifics. On some occasions, we even behave altruistically toward individuals from other species. However, human prosocial behavior is not universal. While some people are strongly prosocial, others are not (Yamagishi et al., 2014).

Studies of prosociality have advanced largely with the employment of experimental economic games in which participants interact with each other over real monetary reward. A number of studies have examined individual differences in prosociality using the experimental economic games. Among them, several have reported genetic influences (for a review see Cesarini et al., 2009b; Navarro, 2009; Ebstein et al., 2010). These studies employed the classic twin method, which employs the fact that identical twin [(monozygotic twin (MZ)] pairs share all their genes, while fraternal twin (dizygotic twin, hereafter DZ) pairs share, on average, half their genes. As both MZ pairs and DZ pairs share a common family environment (i.e., shared environment, C) genetic influences can be inferred if we observe a larger within-pair correlation for MZ pairs than for DZ pairs. Large within-pair correlations for both MZ and DZ pairs suggest the existence of shared environmental influences. Dissimilarity in twin pairs can be attributed to the effects of non-shared environment and error, which are unique to each individual (Neale and Maes, 2002; Plomin et al., 2008).

Wallace et al. (2007) examined heritability of responses to an ultimatum game, which are expected to reflect the participants’ sense of fairness. They reported that 42% of the variation in the responses is genetically influenced. The remaining phenotypic variances (58%) can be explained by non-shared environmental factors. Cesarini et al. (2009a) conducted a modified dictator game that concerns altruism toward unknown people and found that individual variation in responses was explained by 31% of genetic factors and 69% of non-shared environmental influences. Cesarini et al. (2008) have conducted trust game experiments in the US and Sweden. The game measures the level of trust and the level of trustworthiness of the participants. It was shown that a large proportion of phenotypic variance was explained by non-shared environment (trust, 82% for the US, 68% for Sweden; trustworthiness, 71% for the US, 66% for Sweden). Genetics explained a relatively smaller proportion of variance. For trust, genetic influences were estimated to be 10% for the US studies and 20% for the Swedish; for trustworthiness, genetic influences were 17 and 32%, respectively.

The above-mentioned studies have successfully demonstrated genetic and environmental influences on prosociality (i.e., fairness, altruism, trust, and trustworthiness) with experimental economic games. This was consistent with the first of the “three laws of behavior genetics” (Turkheimer, 2000), which states that every human behavioral trait is heritable. To the authors’ knowledge, however, there have been no classic twin studies using the social dilemma scenario.

Formation of the highly cooperative group is one of the distinctive characteristics of human beings (Sober and Wilson, 1998; Fehr and Fischbacher, 2003; Silk and House, 2011). In such cooperative groups, benefit of individuals and benefit of the group often contradict each other. This is called social dilemmas. An example is environmental problems: one can benefit from exploiting environmental resources while damaging the environment as a whole. In social dilemmas, it is better for individuals to free ride on the contribution by other members of the group. However, if everyone free rides, group cooperation will be collapsed (Diamond, 2005).

Large numbers of studies have been conducted to clarify the condition under which group cooperation is maintained. One necessary condition is repeated interaction. As far as the interaction is one-shot, free riding is the best strategy in social dilemmas. When there is a chance of future interaction, cooperation could be a better strategy because it could elicit cooperation from the partners, resulting in a reciprocal exchange of cooperation (Trivers, 1971; Axelrod, 1984; Nowak, 2006).

In the current study, we focus on the etiology of individual differences in N-person social dilemmas where more than three individuals are involved. Preceding studies with twins have used economic games that modeled the dyadic social interaction. However, humans often interact in a larger group. Knowledge of the genetics of cooperative behavior in groups would provide insight into the evolution of human prosociality that was driven by genes. In addition, it would also be useful to understand individual variation in our daily life where N-person social dilemma prevails (tax paying, waste disposal, social loafing, etc.).

We examined how much, and in what way, individual differences in N-person social dilemma responses are heritable using the strategy method employed by Fischbacher et al. (2001). They examined phenotypic individual differences in public goods games, a type of N-person social dilemma. In a public goods game, participants can make a contribution to the group from a pre-endowed amount of money. The sum of the contributions from group members is multiplied by (typically) two, and then divided equally among the members. Fischbacher et al. (2001) used the strategy method: They asked participants to indicate how much they would contribute to the group if (1) they did not know the contribution by other members (unconditional decision), and (2) they did know the contribution by others (conditional decision). Several response patterns were observed for the conditional decisions. The majority of responders were conditional cooperators who increased their contribution as the contributions by others increased. These responses were economically “irrational” because the game was one-shot. Some were free riders who consistently made small contributions regardless of others’ contributions, which was considered economically “rational” decision. Similar patterns have been reported by Kurzban and Houser (2001, 2005) who conducted circular public goods games which examined individual differences in a more interactive setting. Ishii and Kurzban (2008) also reported the similar patterns with a Japanese undergraduate sample.

Based on the first law of behavior genetics and evidence from twin studies on other types of economic games, it was predicted that a proportion of phenotypic individual variances in the game would be explained by genetics; however, the exact heritability estimate was unpredictable. We also examined patterns of change in heritability. It has been shown that there are at least two types of strategies adopted in N-person social dilemmas—free riding and conditional cooperation—that differ in their responses to cooperative others. When others are not cooperative, neither type cooperates. When others are cooperative, conditional cooperators similarly cooperate but free riders do not. In other words, greater cooperativeness by others is linked with greater phenotypic variance. Large phenotypic variance does not necessarily lead to higher heritability because heritability is the proportion of phenotypic variance that can be explained by genetic variance. In fact, heritability is smaller when phenotypic variance is larger if genetic variance remains constant. The level of genetic variation is affected by natural selection; put simply, when a trait is under strong selection pressure genetic variation decreases, leading to a drop in heritability. However, under certain conditions, such as negative frequency dependent selection, environmental homogeneity, and selection-mutation balance, fitness-related genetic variance can be maintained through natural selection (Buss, 1991, 2009; Penke et al., 2007; Hiraishi et al., 2008). We examined the patterns of change in heritability as the level of cooperativeness by others increased. This was made possible by the employment of the strategy method.

Two possibilities were considered. The first was that genetic variance would be constantly low regardless of the cooperativeness of others. Cooperation is always disadvantageous in a one-shot N-person social dilemma. Therefore, nature could have selected out those genetic factors that make organisms cooperative. If this were the case, heritability would be smaller when others were cooperative compared with when they were uncooperative. In other words, this pattern would suggest that it is the environment, not genetics, that makes people “irrationally” cooperate with cooperators in one-shot games. The second possibility was that the increase of phenotypic variance was, at least partly, explained by an increase in genetic variance. The tendency to cooperate with cooperative others could have a fitness advantage in the real world where repeated interaction is usual. That means the genetic factors are maintained through natural selection. Those genetic factors may lead organisms to be cooperative with cooperators even in a one-shot interaction. If this were the case, heritability would be equal or greater when others were cooperative. To test these two possibilities, we conducted two social dilemma games with twin participants (Study 1 and Study 2). Study 1 was an on-site group experiment that followed the procedures of Fischbacher et al. (2001). Participants could meet other participants even though they did not know who exactly were their game partners. Study 2 was a web-based experiment where participants’ anonymity was highly confirmed because they interacted via Internet.

One of the weaknesses of the strategy method is that it only measures strategies. Even conditional cooperators will behave uncooperatively if others are not cooperative at all. Actual behavior is determined by the interaction between an individual’s strategy and the social environment, which consists of decisions by others. Even in a society composed of both conditional cooperators and free riders, members may choose not to cooperate if group cooperation is low; therefore, even if heritability is found for some strategies, their payoffs from the social dilemma game may not be as heritable. To observe the effect of strategies on behavior and payoffs from the social dilemma game, we conducted Monte Carlo simulations using strategies registered by twin participants (Study 3). In this way, we computed twin participants’ expected payoffs from the game and examined genetic influences on them. This approach enabled us to run “virtual” iterated games. As mentioned above, cooperativeness toward cooperators is beneficial only when repeated interaction is expected. By changing the number of iterations, we examined the influences of strategies and genetic factors on expected payoffs from the games with shorter and longer repeated interaction.

## Study 1: Group Experiment

In Study 1, we conducted a public goods game, a type of N-person social dilemma, with twin participants. We followed the procedures employed by Fischbacher et al. (2001), who conducted a public goods game using the strategy method with university students. The experiment was conducted at a university campus in a group setting.

### Method

#### Participants

The Keio Twin Study (KTS) recruited twin participants, aged 14–30 years, through population-based registries in some parts of the Tokyo area in 1998–2002. Participation in the project was voluntary, and of 6,000 pairs of twins to whom we sent letters approximately 1,000 pairs agreed to participate. Zygosity of the registrants was initially determined using a questionnaire consisting of three questions about twins’ physical resemblance (e.g., *As a toddler, were you and your twin “as alike as two peas in a pod”?*) demonstrated to have 93.2% accuracy (Ooki et al., 1993). For twin pairs from whom DNA information was available through blood or buccal smears, zygosity was diagnosed by examining gene polymorphisms. The KTS recruited new registrants in 2007 and 2009 and now has approximately 2,000 pairs of twin registrants (for a detailed description of the KTS, see Ando et al., 2013).

In September 2006, the KTS registrants were asked to attend an on-campus session held at Keio University, where we conducted a computer-assisted psychological experiment, the public goods game experiment, and finally a questionnaire survey, which asked about personality, eating, and sexual behaviors. Twins were asked to participate in the session as a pair; however, some co-twins failed to attend. As a result, 317 twins (225 female and 92 male twins) participated in the session. Participants were paid ¥5,000 excluding tax (∼US$60) for attending and participating in the study. In addition, monetary reward were paid according to participant outcomes on the public goods game experiment.

#### Procedure

The experiment was conducted in a campus conference hall with the capacity to hold 116 individuals. We ran 10 experimental sessions, each of which had 15–41 participants (*M* = 31.8, SD = 10.1). Twin siblings participated in the same session and co-twins were seated on opposite sides of the room. At the start of the on-campus session, the information regarding the study was provided to the participants and informed consent was obtained.

The game instructions were presented on a screen at the front of the room and read aloud by an experimenter. The same instructions were presented on a laptop computer in front of each participant. Upon reading the instructions, participants were required to answer four control questions on the laptop to check their understanding of the game structure. Only those who passed the control questions filled in a response sheet on which they wrote their game decisions. If participants had difficulty understanding the instructions, they could direct questions to the experimental assistants who were told not to use the words “cooperation,” “defection,” or “contribution” in their explanations. Participants were asked not to talk to each other during the experiment.

The participants were told that they would be grouped with three other participants in the room. It was emphasized that group membership would be kept anonymous. Each participant was given 20 points at the outset. They could contribute as many/few points as they wanted to the group. To avoid unnecessary implications, we used the word “invest” instead of “contribute” to explain the procedure. The aggregate investment of the four members of each group was multiplied by 1.6 and distributed equally among members. The sum of the points gained from the investments and the points retained was the outcome for the participants. Each point was converted to ¥20 (about $0.20).

Participants were asked to make two kinds of decisions in the public goods game. One was the unconditional contribution: how many points they would invest if they did not know what others had invested. We named these UC decisions. The other was the conditional contribution: how many points participants would invest if they knew the average investment by the others was 1, 2,... 20 points. We named these C1, C2, … C20 decisions, respectively. Participants were told that for three group members, the UC decisions would be used to calculate the outcome. For the remaining member, the conditional decision was used according to the average UC decision by the other three. After making their decisions, participants placed their response sheet in an envelope that was retrieved by an assistant who then gave them the booklet containing the personality questionnaire and the eating and sexual behavior survey. It took about 30 min to complete the public goods game. After participants had completed the questionnaire, they were informed of their outcome from the public goods game. The sum of the attendance fee and the game outcome was paid to participants’ bank accounts within one month. All procedures were explained to participants before they made their decisions. Experimental procedures for all three studies were approved by the ethics committee at the Faculty of Letters, Keio University.

### Results

#### Simple Statistics

Some of the participants required extensive help from the assistants to complete the control questions. Removing those 21 participants, data from 296 individuals were analyzed (209 females and 87 males, 213 MZ twins, 61 same sex DZ twins, and 22 opposite sex DZ twins). Age ranged from 18 to 37 years (*M* = 25.42, SD = 4.27).

Mean conditional decisions increased in accordance with the investment by other group members (**Table 1**). We regressed sample mean decisions on others’ investment. The regression coefficient was positive and highly significant (*b^∗^* = 0.986, *p* < 0.001; *R^2^* = 0.973, *p* < 0.001). This indicated that, on average, participants displayed a conditional cooperation strategy. We also observed that the variances (SD) of the decisions became larger as the contributions by others increased (i.e., C20 decision). The regression of SD on others’ investment showed strong positive relationship (*b^∗^* = 0.921, *p* < 0.001; *R^2^* = 0.849, *p* < 0.001). This showed that the degree of conditionality was not monotonic, especially when others were behaving cooperatively. In fact, 47 (15.8%) participants did not change their investment through C1–C20. These differences in the pattern contributed to the larger variances in the greater contribution settings.

**Table 1:** Mean and SD of game decisions in Study 1.

Decisions	*M*	SD
C1	2.38	4.85
C2	2.82	4.59
C3	3.25	4.54
C4	3.87	4.43
C5	4.35	4.45
C6	4.69	4.35
C7	5.09	4.37
C8	5.56	4.53
C9	5.99	4.75
C10	6.73	5.24
C11	7.11	5.59
C12	7.35	5.79
C13	7.51	6.02
C14	7.76	6.28
C15	8.13	6.68
C16	8.32	7.06
C17	8.28	7.33
C18	8.61	7.70
C19	9.01	8.11
C20	9.73	8.72

UC (Unconditional)	7.25	5.74

LC (C1–C5)	3.34	4.31
MLC (C6–C10)	5.61	4.27
MHC (C11–C15)	7.57	5.81
HC (C16–C20)	8.79	7.38

LC, lowest conditional; MLC, middle-low C; MHC, middle-high C; HC, highest C

#### Genetic Analysis

We conducted genetic analyses for 98 MZ pairs and 29 DZ pairs of which both co-twins had participated in the study. To qualitatively analyze the genetic and environmental influences, we calculated four scores from the conditional decisions and subjected them to genetic analyses: lowest C (LC) scores (average of C1 to C5 decisions), middle-low C (MLC) scores (average of C6–C10), middle-high C (MHC) scores (average of C11–C15), and highest C (HC) scores (average of C16–C20). **Table 2** shows the within-pair intraclass correlations for the four conditional scores and UC scores for MZ pairs and DZ pairs. The correlation coefficients were small even for MZ pairs, suggesting relatively small genetic influences. The differences between MZ pair correlations and DZ pair correlations were largest for the HC scores, which suggests that genetic influences were greater for decisions made in higher cooperativeness settings.

**Table 2:** Within-pair intraclass correlations and 95% credible intervals for decision scores in Study 1.

	MZ	95% CI		DZ	95% CI	
UC	0.05	[0.15,	0.25]	-0.45	[-0.69,	-0.10]
LC	0.09	[-0.11,	0.28]	-0.07	[-0.41,	0.30]
MLC	0.12	[-0.08,	0.31]	0.15	[-0.22,	0.48]
MHC	0.14	[-0.06,	0.33]	0.07	[-0.29,	0.42]
HC	0.21	[0.01,	0.39]	0.02	[-0.34,	0.38]

MZ, monozygotic twin; DZ, dizygotic twin; CI, credible interval; UC, unconditional; LC, lowest conditional; MLC, middle-low C; MHC, middle-high C; HC, highest C.

To further analyze genetic and environmental influences, we conducted univariate genetic model-fitting analyses using Markov chain Monte Carlo (MCMC) algorithms (van den Berg et al., 2006). For each decision score, we constructed a Bayesian ACE model, in which the influences of additive genetic factors (A), familial shared environmental factors (C) and non-shared environmental factors were assumed to explain the variances and covariances of the twin data. Specifically, the model was:

Yij=mu+Ali+A2i+Ci+Eij

where the observed score of an individual i from a family j (Yij, e.g., UC score) was a sum of the population mean (mu), additive genetic factors (A1i and A2i), familial shared environment factors (Ci), and non-shared environment factors and error (Eij). For MZ pairs, both the A1i and A2i were shared among the twin siblings whereas for DZ pairs, A1i was shared while A2i was unique to each twin. As the variances of A1i and A2i were set to be equal, the genetic covariance in MZ pairs was twice as large as in DZ pairs. In making the Bayesian inference with MCMC algorithms, we assumed no prior information about the parameter estimates and therefore chose a relatively non-informative uniform distribution for the variance components. That is, A1i ∼ U (0, 50), A2i ∼ U (0, 50), Ci ∼ U (0, 100), Eij ∼ U (0, 100), and mu ∼ N (0, 100). Therefore, the joint posterior distribution is mainly determined by the likelihood. With the model, we estimated the posterior distribution of the additive genetic (A), shared environmental (C), and non-shared environmental factors (E). It should be noted that E included measurement error.

The models were implemented in WinBUGS software, which ran MCMC sampling with the R2WinBUGS package for R software. For each model, three Markov chains were produced, each of which had 100,000 iterations that were thinned by 100. The first 10,000 iterations were discarded as a “burn-in” period. We checked if the models reached the stationary posterior distribution using the Gelman and Rubin (G-R) statistic (values closer to 1 indicate convergence). For all chains, the G-R statistics were less than 1.1.

**Table 3** shows the parameter estimates for each score. The mean A estimation was largest for the HC score (12.6%), followed by the MHC (9.8%) and the MLC scores (9.3%). For the LC and UC scores, the mean A estimations were quite small (7.8 and 6.3%, respectively). Similarly, mean C estimations were larger for HC, MHC, and MLC scores (9.6, 9.8, and 9.1% respectively) compared with the LC and UC scores (5.0 and 4.5%, respectively).

**Table 3:** Genetic and environmental factor estimations in Bayesian ACE models in Study 1.

Scores	G-R	A	95% CI		C	95% CI		E	95% CI	
UC	1.02	0.06	[0.00,	0.18]	0.05	[0.00,	0.16]	0.89	[0.76,	0.98]
LC	1.00	0.08	[0.00,	0.22]	0.07	[0.00,	0.20]	0.85	[0.71,	0.97]
MLC	1.02	0.09	[0.00,	0.25]	0.09	[0.00,	0.25]	0.82	[0.66,	0.95]
MHC	1.02	0.10	[0.00,	0.26]	0.09	[0.00,	0.25]	0.81	[0.66,	0.95]
HC	1.01	0.13	[0.01,	0.31]	0.10	[0.00,	0.25]	0.78	[0.63,	0.93]

Mean and 95% credible intervals of parameter estimates are shown. A denotes additive genetic factor; C, shared environmental factor; E, non-shared environmental factor and error; G-R, Gelman and Rubin statistics; LC, lowest conditional; MLC, middle-low C; MHC, middle-high C; HC, highest C.

### Discussion

Our findings replicated those of Fischbacher et al. (2001) and showed phenotypic individual differences of our twin participants that included free rider and conditional cooperator strategies.

For all decision scores, the majority of phenotypic variances (variances in scores) were explained by non-shared environmental factors and error (E). Because the experiment employed a one-shot economic game, moderately large measurement error might have contributed to the phenotypic variances. We should be cautious not to overestimate the influences of non-shared environments such as school, peers, and society.

We observed larger influences of additive genetic factors (A) on decisions when the other members were more cooperative. The A for more cooperative settings (MHC and HC scores) were twice as large as that for the least cooperative setting (LC score). This suggests that genetic factors that make organisms conditionally cooperative are maintained through natural selection. However, in the current study, there was limited anonymity among participants. Even though group membership was not revealed, participants could interact with each other before and after the experiment. This could have affected the results in ways that we have yet to identify. Another problem was that a part of the participants (29 out of 317, 6.6%) had difficulty in understanding the game structure. We addressed these problems in the next experiment.

## Study 2: Web-Based Experiment

For Study 2, we implemented a web-based strategy method experiment. Participants logged in to the experiment website and registered their decisions via the Internet, which ensured anonymity. In addition, the website interactively showed the expected payoffs as participants input their decisions, helping them to understand the game structure.

### Method

#### Participants

We sent recruitment letters to about 3,000 KTS registrants in November 2009. From these, 282 twins participated in the study (190 females and 92 males). Ages ranged from 18 to 32 years (*M* = 22.69, SD = 3.60). There were 199 MZ twins, 52 same sex DZ twins, and 31 opposite sex DZ twins. Among them, 73 had participated in Study 1.

#### Procedure

We invited participants to access the experiment website (www.futago-labo.net) with a letter explaining the web survey system. On the website, participants first read the instructions for the public goods game experiment and completed some trial sessions. Participants then read the informed consent information. Those who agreed to the informed consent logged in to the response page with an ID and password provided with the invitation letter. On the response page, participants registered their unconditional decisions (UC) and conditional decisions (C0–C20).

There were several differences between this study and Study 1. First, participants registered their responses via the Internet. They individually read the instructions on a web browser and registered their decisions, and the surrounding environment was not controlled at all. This was very different from Study 1, in which participants came to a university campus, were seated in a quiet room facing the experimenter and assistants, and met other participants. As even subtle cues of the existence of another person, such as eye-like paintings, can influence behavior in experimental economic games (Haley and Fessler, 2005; Burnham et al., 2007; Oda et al., 2011; Nettle et al., 2013), the difference between the group experiment and the web experiment was enormous. Second, as participants recorded a conditional decision on the response page, the expected payoffs for the participants and other members were explicitly indicated. Third, the game rule was changed so that the aggregate contribution was doubled and distributed equally among the four group members. In other words, the return rate of the investment was larger (0.5 times for Study 2 and 0.4 times for Study 1). Fourth, we asked the participants to register C0 decisions, which we failed to collect in Study 1. Fifth, there was no show-up fee for Study 2. The second and third changes were intended to make it easier for participants to understand the game structure.

Registered responses were randomly grouped and game payoffs computed. The results were sent to the participants via postal mail. By the same mail, participants were asked to send back their bank account information, so that payoffs could be transferred to them (1 point = ¥20). All the procedures were explained before participants logged in to the response webpage.

In preparation for the experiment with twin participants, we conducted a preliminary experiment with undergraduates (*n* = 37; Hiraishi, unpublished data). The results were generally consistent with Fischbacher et al. (2001) study; we observed the two major strategies of conditional cooperation (*n* = 17) and free riding (*n* = 16).

The experimental procedures were approved by the ethics committee at the Faculty of Letters, Keio University.

### Results

#### Simple Statistics

We found that as the contribution by others increased, both the mean contribution decisions and the variances of the conditional decisions increased (**Table 4**). Regression of mean contribution decisions on others’ contribution showed significant positive relationship (*b^∗^* = 0.996, *p* < 0.001; *R^2^* = 0.992, *p* < 0.001). The same applied to *SD* as well (*b^∗^* = 0.982, *p* < 0.001; *R^2^* = 0.963, *p* < 0.001). While the majority of participants (*n* = 123, 43.6%) adopted a conditional cooperation strategy, 70 participants (24.8%) adopted a free rider strategy, contributing zero points through C0–C20 decisions.

**Table 4:** Mean contributions in Study 2.

Study 2	*M*	SD
C0	1.00	3.37
C1	1.67	3.64
C2	2.19	3.70
C3	2.53	3.55
C4	3.01	3.75
C5	3.63	4.04
C6	4.15	4.23
C7	4.52	4.48
C8	5.06	4.79
C9	5.57	5.08
C10	6.22	5.21
C11	6.84	5.59
C12	7.26	5.84
C13	7.71	6.18
C14	7.89	6.49
C15	8.43	6.98
C16	8.80	7.37
C17	8.89	7.76
C18	9.50	8.13
C19	9.74	8.58
C20	9.98	9.14

UC2	7.03	6.21
LC2 (C0–C6)	2.60	3.33
MC2 (C7–C13)	6.17	4.98
HC2 (C14–C20)	9.03	7.48

UC, unconditional; LC, lowest conditional; MC, medium C; HC, highest C scores in Study 2.

Because we had 21 conditional decision scores from each participant (C0–C20), we computed three conditional decision scores. They represented low contribution in Study 2 (LC2 scores; average of C0–C6 decisions), medium contribution in Study 2 (MC2) scores (average of C7–C13), and high contribution in Study 2 (HC2) scores (average of C14–C20). As there were no significant differences between MLC (C6–C10) scores and MHC scores (C11–C15) in Study 1, we decided to merge the MLC and MHC categories to obtain three, rather than four, overall scores.

#### Comparison of Repeaters, Non-Repeaters, and First-Comers

Seventy-three participants were repeaters from Study 1. We compared the repeaters’ decisions in Study 1 with those of non-repeaters (those who participated only in Study 1). Repeaters had significantly lower LC and MLC scores (LC score, Wilcoxon test, *W* = 9582.5, *p* < 0.05; MLC score, *W* = 9536.5, *p* < 0.05). There were no significant differences in UC, MHC, and HC scores for repeaters and non-repeaters. Next, we compared repeaters (*n* = 73) and newcomers (*n* = 209) on their decisions in Study 2. Repeaters contributed significantly less than newcomers for all categories (UC2 scores, *W* = 9441.5, *p* < 0.01; LC2 scores, *W* = 9330.5, *p* < 0.01; MC2 scores, *W* = 9537, *p* < 0.01; HC2 scores, *W* = 9271.5, *p* < 0.01).

#### Correlations Between Repeaters’ Decisions in Study 1 and Study 2

We computed correlation coefficients for repeaters’ decision scores in Study 1 and Study 2. There were significant correlations for most of the decision scores for Studies 1 and 2 (**Table 5**), except that UC2 scores were significantly correlated only with UC scores in Study 1, and Study 1 HC scores were not significantly correlated with any Study 2 scores, though LC2, MC2, and HC2 scores approached significance (*p* < 0.1).

**Table 5:** Spearman correlation coefficients for Study 1 and Study 2 (*n* = 73).

*rho*	Study 2			
Study 1	UC2	LC2	MC2	HC2
UC	0.24^∗^	0.40^∗∗∗^	0.44^∗∗∗^	0.40^∗∗∗^
LC	0.10	0.25^∗^	0.21†	0.21†
MLC	0.19	0.30^∗^	0.31^∗∗^	0.28^∗^
MHC	0.11	0.19	0.25^∗^	0.30^∗^
HC	0.13	0.23†	0.22†	0.23†

*In Study 1, UC, unconditional; LC, lowest conditional; MLC, middle-low C; MHC, middle-high C; HC, highest C. In Study 2, UC, unconditional; LC, lowest conditional; MC, medium C; HC, highest C.*

*†*p* < 0.1, ^∗^*p* < 0.05, ^∗∗^*p* < 0.01, ^∗∗∗^*p* < 0.001.*

#### Genetic Analysis

We conducted univariate genetic analyses using data from 64 MZ pairs and 22 DZ pairs of which both co-twins had participated in the study. Among them, 21 MZ pairs and one DZ pair were repeaters from Study 1. The statistical procedures were the same as in Study 1.

We found that the additive genetic influences (A) increased as the contribution by others increased. Mean parameter estimates for additive genetic factors were 16, 21, and 26% for LC2, MC2, and HC2 scores, respectively (**Table 6**). There was a reduced relative contribution by non-shared environmental influences; 73, 64, and 59% for LC2, MC2, and HC2 scores, respectively. For unconditional decisions (UC2 scores), the parameter estimate for the additive genetic factor was similar to that for MC2 scores (22 and 21%, respectively).

**Table 6:** Genetic and environmental factor estimations in Bayesian ACE models in Study 2 with non-informative prioris.

Score	G-R	A	95% CI		C	95% CI		E	95% CI	
UC2	1.02	0.22	[0.02	0.44]	0.10	[0.00	0.30]	0.68	[0.49	0.88]
LC2	1.03	0.16	[0.01	0.38]	0.11	[0.01	0.30]	0.73	[0.54	0.92]
MC2	1.02	0.21	[0.01	0.45]	0.15	[0.01	0.38]	0.64	[0.47	0.83]
HC2	1.01	0.26	[0.02	0.52]	0.15	[0.01	0.39]	0.59	[0.41	0.79]

Mean and 95% credible intervals of parameter estimates are shown. A denotes additive genetic factor; C, shared environmental factor; E, non-shared environmental factor and error; G-R, Gelman and Rubin statistics; UC, unconditional; LC, lowest conditional; MC, medium C; HC, highest C.

We also conducted genetic analyses using information from Study 1 as priors. Specifically, we took the means and variances of A, C, E, and mu (population mean) parameters estimates in Study 1 and used them as parameters for prior normal distribution for A, C, E, and mu in Study 2, respectively. The information from LC scores was used for LC2 analysis. The information from MLC and MHC scores were, separately, used for MC2 analysis. The information from HC scores was used for HC2 analysis. We found that the additive genetic influences were smaller with the informative priors than with non-informative priors, reflecting the fact that we had smaller A estimates in Study 1. Still, we found the same pattern: Genetic influences increased as the contribution by others increased (**Table 7**).

**Table 7:** Genetic and environmental factor estimations in Bayesian ACE models in Study 2 with prior informative from Study 1.

Score	G-R	A	95% CI		C	95% CI		E	95% CI	
UC2	1.00	0.08	[0.02	0.15]	0.06	[0.01	0.11]	0.86	[0.79	0.92]
LC2	1.00	0.06	[0.00	0.15]	0.05	[0.00	0.14]	0.89	[0.78	0.98]
MC2(1)	1.00	0.13	[0.04	0.21]	0.12	[0.03	0.20]	0.75	[0.66	0.85]
MC2(2)	1.00	0.09	[0.01	0.17]	0.08	[0.01	0.16]	0.83	[0.74	0.92]
HC2	1.00	0.14	[0.07	0.20]	0.10	[0.04	0.16]	0.76	[0.69	0.84]

Mean and 95% credible intervals of parameter estimates are shown. A denotes additive genetic factor; C, shared environmental factor; E, non-shared environmental factor and error; G-R, Gelman and Rubin statistics; UC, unconditional; LC, lowest conditional; MC, medium C; HC, highest C. MC(1) indicates estimates with MLC information as priors. MC(2) indicates estimates with MHC information as priors.

### Discussion

The findings generally replicated those of Study 1. Most participants adopted the conditional cooperating strategy. However, some participants adopted the free rider strategy, constantly making zero contribution decisions. Genetic analyses showed that non-shared environmental factors explained more than half of the phenotypic variances in all decision scores. Additive genetic influences were greater for decisions made when other members were making larger contributions.

Even though Study 2 featured significantly different procedures from Study 1, the patterns and etiology of individual differences were similar. Some of the participants in Study 2 were repeaters from Study 1 (26%, 73 out of 282). This may partly explain the similar results of the two studies. The data show that there were significant within-participants correlations in decisions between the two studies. In addition, the repeaters had certain characteristics. They were less cooperative than non-repeaters in Study 1 and newcomers in Study 2. The percentages of the free rider strategy in Study 2 (zero contribution for all conditional decisions) were 21% for newcomers and 37% for repeaters (*Z* = 2.64, *p* < 0.01). These confounding factors indicate that the generality of the results may be limited.

From a different perspective, the correlations between Study 1 and Study 2 set the expedient upper limit for the heritability estimates. This is because the correlations indicated the strength of relationships between the same individuals separated by a certain time interval (about 3 years) while the heritability estimates indicated relationships between different individuals sharing genetics and environments. As the calculation of decision scores differed between the two studies, the upper limit was considered expediential. However, it was remarkable to see that the heritability estimates were close to their expedient upper limit. For instance, the mean heritability estimate for UC2 scores was 0.22 while the correlation between UC and UC2 scores was 0.24. For HC2 scores, the mean heritability estimate was 0.26 and the correlation between HC and HC2 scores was 0.23, exceeding its expedient upper limit. This suggested that the apparently small heritability estimates were partly attributable to weak measurement reliability. The ratios for heritability to the expedient upper limit were greater for the decision scores under the highly cooperative group setting. The ratios were 0.64, 0.68, 0.83, and 1.13 for LC2:LC, MC2:LMH, MC2:MHC, and HC2:HC, respectively. These again suggested that additive genetic influences were greater for the decisions in situations where other members were making larger contributions.

## Study 3: Monte Carlo Simulation

In our first two studies, we examined heritability of individual differences in strategies on public goods games. However, because of the weakness of the strategy method, differences in strategies do not necessarily lead to differences in behavior and differences in game outcomes. Therefore, we simulated participants’ behavior and expected game outcomes using Monte Carlo simulations.

The game payoff for each participant is a function of the individual’s decision and the decisions of the other members. An examination of all possible combinations of participants would reveal each participant’s expected payoff. However, the number of possible combinations could be quite large and practically impossible to compute. For example, drawing four individuals from the 282 participants (Study 2) makes 257,932,710 combinations. Therefore, we employed Monte Carlo simulation to create a large number of groups consisting of four individuals randomly selected from the 282 participants. We conducted seven simulations. The first simulation calculated the payoffs in the same way as for Study 2; there was no iteration of games. The remaining six simulations were “virtually” iterated games. After obtaining expected payoffs for each individual, we estimated genetic and environmental influences on the payoffs.

### Method

#### Monte Carlo Simulation with One-Shot Games

We randomly sampled four individuals from the 282 Study 2 participants. As we failed to collect conditional decisions when others were making zero contributions, we could not use data from Study 1. We calculated the game payoffs for these four individuals in the same way as for Study 2. That is, one individual was randomly chosen as a conditional decision maker while the remaining three individuals were designated unconditional decision makers. Each individual was endowed with 20 points at the outset. We took the UC2 decisions by the unconditional decision makers. Then, according to the average contribution by the unconditional decision makers, the conditional decision maker’s contribution was decided based on his/her conditional decisions in Study 2. The aggregate contribution was doubled and distributed equally among the four individuals. For each individual, the outcome was the sum of earnings from the group contribution and the leftover money. We sampled 250,000 groups. For each individual, we computed the average payoff as an unconditional decision maker and the average payoff as a conditional decision maker. The sum of the two payoffs constituted the individual’s total estimated payoffs.

#### Monte Carlo Simulation with Virtually Iterated Games

We randomly sampled 10,000 groups composed of four individuals from the 282 participants in Study 2. For the first round, their contributions were made by their unconditional decisions in Study 2. For the following rounds, their contributions were chosen based on the contributions by others in the preceding round and the conditional decisions in Study 2. For instance, if the other members (e.g., L, M, N) contributed, on average, 10 points in the first round, the contribution of the fourth member (P) in the second round would be his C10 decision in Study 2. The same was true for L, M, and N members. The number of iterations was prefixed 2, 5, 10, 20, 50, and 100 iterations. For each iterated game, we sampled 100,000 groups.

### Results

#### Monte Carlo Simulation with One-Shot Games

Each participant was chosen as an unconditional decision maker, on average, 2664.57 times (SD = 57.74). The mean number of times chosen as a conditional decision maker was 891.52 (SD = 29.16). The mean payoff as an unconditional decision maker was 25.91 points (SD = 2.60; **Table 8**). The payoff was strongly negatively correlated with the UC2 (*rho* = -0.971, *p* < 0.0001; **Table 9**, first column), indicating that cooperativeness led to lesser payoffs. The mean payoff as a conditional decision maker was 28.17 points (SD = 2.07; **Table 8**). The payoffs were strongly negatively correlated with conditional decision scores in Study 2 (**Table 9**, second column), again suggesting that those who were more cooperative earned less. The difference between the payoff as an unconditional decision maker and that as a conditional decision maker was significant, indicating that the payoffs were larger for conditional decision makers [*t*(281) = 16.137, *p* < 0.0001].

**Table 8:** Mean, SD, and correlation with Study 2 decisions for payoffs in Study 3.

	Uncond.	Cond.	It. = 2	It. = 5	It. = 10	It. = 20	It. = 50	It. = 100
*M*	25.91	28.17	25.88	24.50	23.83	23.53	23.33	23.27
SD	2.60	2.07	1.60	0.85	0.61	0.55	0.55	0.53

Uncond. denotes payoffs for unconditional decision makers in simulations without iteration; Cond., payoffs for conditional decision makers in simulations without iteration; It., the number of iterations (It. = 2 through 100, denoting simulations with 2, 5,10, 20, 50, and 100 iterations).

**Table 9:** Correlations between Study 2 decisions and payoffs in Study 3.

Scores	Uncond.	Cond.	It. = 2	It. = 5	It. = 10	It. = 20	It. = 50	It. = 100
UC2	-0.971	-0.535	-0.836	-0.711	-0.510	*0.136*	0.302	0.340
LC2	-0.520	-0.902	-0.828	-0.925	-0.854	-0.271	-*0.034*	*0.047*
MC2	-0.549	-0.959	-0.871	-0.865	-0.707	-*0.020*	0.246	0.331
HC2	-0.539	-0.851	-0.790	-0.752	-0.582	*0.108*	0.385	0.438

All correlation coefficients were significant (*p* < 0.0001) except for those indicated in italics. Uncond. denotes payoffs for unconditional decision makers in simulations without iteration; Cond., payoffs for conditional decision makers in simulations without iteration; It., the number of iterations (It. = 2 through 100, denoting simulations with 2, 5,10, 20, 50, and 100 iterations); UC, unconditional; LC, lowest conditional; MC, medium C; HC, highest C.

We conducted univariate genetic analyses in the same manner as in Study 2 for the payoffs (**Table 10**). The results for unconditional decision maker payoffs were almost identical to those for unconditional decision scores in Study 2. The mean estimate of additive genetic factors was 22% while most of the phenotypic variances were explained by non-shared environmental factors (68%).

**Table 10:** Univariate genetic analyses for payoffs in Monte Carlo simulations.

	G-R	A	95% CI		C	95% CI		E	95% CI	
Uncond.	1.01	0.22	[0.02,	0.46]	0.10	[0.00,	0.30]	0.68	[0.48,	0.88]
Cond.	1.03	0.19	[0.01,	0.43]	0.14	[0.01,	0.37]	0.67	[0.49,	0.86]
It. = 2	1.01	0.30	[0.04,	0.55]	0.12	[0.00,	0.35]	0.58	[0.39,	0.79]
It. = 5	1.01	0.21	[0.01,	0.44]	0.12	[0.00,	0.33]	0.68	[0.49,	0.87]
It. = 10	1.01	0.15	[0.01,	0.39]	0.11	[0.01,	0.30]	0.74	[0.54,	0.92]
It. = 20	1.02	0.15	[0.01,	0.37]	0.11	[0.01,	0.29]	0.75	[0.54,	0.94]
It. = 50	1.01	0.17	[0.01,	0.42]	0.14	[0.01,	0.34]	0.69	[0.49,	0.88]
It. = 100	1.03	0.19	[0.01,	0.44]	0.13	[0.01,	0.34]	0.68	[0.48,	0.88]

Mean parameter estimates with their 95% credible intervals for ACE models are presented, where A denotes additive genetic factors, C, familiarly shared environmental factors, and E, familiarly non-shared environmental factors. Uncond., unconditional decision makers in simulation without iteration; Cond., conditional decision makers in simulation without iteration; It., the number of iterations (It. = 2 through 100, denoting simulations with 2, 5, 10, 20, 50, and 100 iterations); G-R, Gelman and Rubin statistics.

The parameter estimates for conditional decision makers indicated that most of the phenotypic variances were explained by non-shared environmental factors (67%) while additive genetic factors explained 19% and shared environmental factors explained 14%.

#### Monte Carlo Simulation with Iterated Games

**FIGURE 1:**
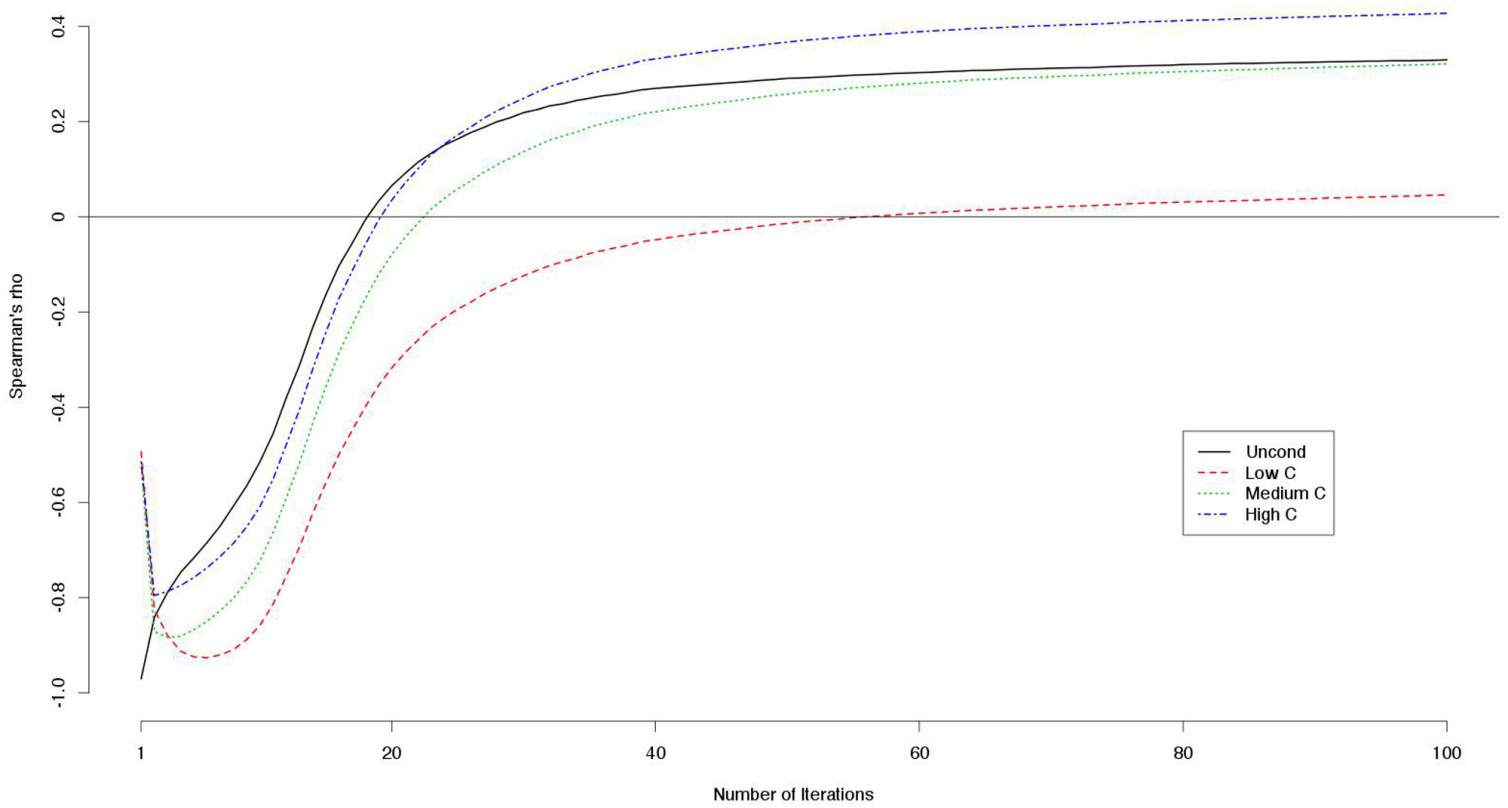
**Correlations (Spearman’s *rho*) between decision scores and outcomes on the simulated games with iterations**.

The mean payoffs per round were larger when the numbers of iterations were smaller (**Table 8**). Correlations between decision scores and payoffs showed particular patterns (**Table 9**). The correlation coefficients were negative when the number of iterations was small, but they were positive when the number of iterations was large. We visualized the patterns using data from another simulation with 100 iterations. For each round, we computed the correlations between the decision scores and the payoffs up to that round. For instance, we took the sum of the payoff from the first 10 rounds, and divided it by 10. This gave us an estimate of the average payoff per round of the games with 10 iterations. In this way, we computed the correlations between the decision scores and the outcomes per round for games with 1–100 iterations (**Figure 1**). We found that for the games with smaller iterations, the decisions and the outcomes were strongly negatively correlated. However, the absolute correlations became smaller as the number of iterations grew. With even larger numbers of iterations, the correlations became positive.

**FIGURE 2:**
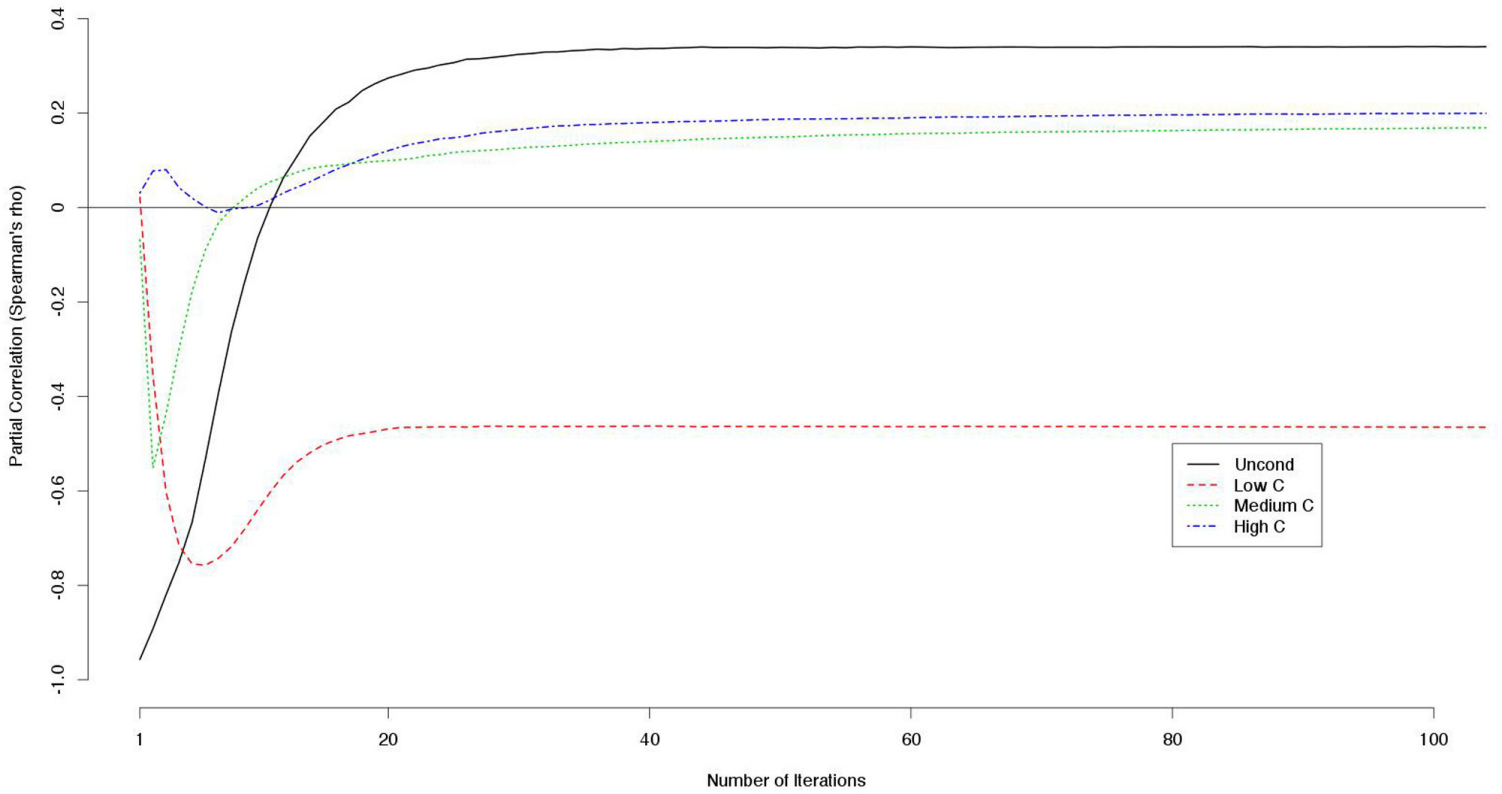
**Partial correlations between decision scores and outcomes controlling for the other decision scores (e.g., partial correlations between UC2 score and the outcome controlling for the LC2, MC2, and HC2 scores are indicated)**.

Because the decision scores were correlated with each other, we computed the partial correlation between a decision score (e.g., a UC2 score) and the payoff controlling for the other decision scores (e.g., LC2, MC2, and HC2 scores). The LC2 scores constantly correlated negatively with the outcome while the other scores correlated positively with larger numbers of iterations (**Figure 2**).

Univariate genetic analyses were conducted in the same manner as in Study 1 and Study 2. For all five simulations, most of the phenotypic variances were explained by non-shared environmental factors. As the number of iterations increased, the strength of additive genetic factors decreased as long as there were less than 20 iterations. For instance, the mean estimate of additive genetic factors was 0.30 for games with two iterations and 0.15 for games with 10 and 20 iterations. However, with larger numbers of iterations (50 or 100 times), the strength of additive genetic factors increased (**Table 10**). For games with 100 iterations, the mean estimate of additive genetic factors was 0.19.

### Discussion

For the simulated one-shot game, there were strong phenotypic correlations between decision scores and simulated payoffs. For unconditional decision maker payoffs, phenotypic correlations between the UC2 scores and the simulated payoffs were quite large (*rho* = –0.97), suggesting that the genetic influences on the payoffs were equivalent to the genetic influences on the UC2 scores. Because we sampled a large number of groups (250,000), the payoffs for each decision maker approached their expected payoffs, which explains the strong correlations between their decisions and payoffs.

The payoffs for conditional decision makers, whose conditional decisions were used in the simulation, were strongly correlated with conditional decision scores (LC2, MC2, and HC2), especially with MC2 scores (*rho* = 0.95). The magnitude of genetic influences on the payoffs was equivalent to that of MC2 scores in Study 2. This relates to the fact that the mean UC2 was 7 points. As the conditional decision makers made their decisions based on the mean unconditional contribution by other members, they were most likely to have taken the C7 decision during the simulation, whereas the MC2 score was the mean of C7–C13 scores.

For the simulation with iterated games, genetic influences first decreased then increased as the number of iterations grew. Specifically, for games with 2, 5, 10, and 20 iterations, the genetic influences decreased from 29.6% (iteration = 2) to 14.5% (iteration = 20). For games with 50 iterations, the genetic influences recovered to 17.4%. This trend continued for games with 100 iterations, which showed 19.1% of genetic influences. This pattern can be explained as follows.

For the games with smaller iterations, the decision scores and payoffs were strongly negatively correlated. In other words, cooperativeness had negative effects on the payoffs. This was because cooperators were likely to be exploited by non-cooperators. Because decision scores and payoffs were correlated, it is not surprising that the payoffs were genetically influenced. However, as the number of iterations got larger, the negative correlations between the decisions and the payoffs became weaker. This was possibly because cooperators could benefit from repeated cooperation with other cooperators. The benefits increased as the number of iterations grew, compensating for the exploitation by non-cooperators. As a result, for games with 20 iterations, the correlations between the decision scores and payoffs approached almost zero because the negative and positive effects were balanced. For these, phenotypic variance was mostly explained by chance factors included in the E parameter. This explains the first decrease in the genetic influences. The same process can explain the recovery of genetic influences with larger numbers of iterations. For these, the benefits of repeated cooperation outperformed the loss imposed by the exploitation, leading the payoffs to be positively correlated with the decisions.

Notably, the correlation between LC2 scores and payoffs was constantly negative and never approached zero when controlling for the other decision scores (**Figure 2**). This means that cooperativeness toward non-cooperators had negative effects on the payoffs regardless of the number of iterations.

## General Discussion

We conducted two public goods game experiments with the classic twin method (Study 1 and Study 2). Most of the individual differences in the games were explained by non-shared environmental factors and errors (E). The genetic influences were relatively small, explaining 10–40% of the phenotypic variances. It was noticeable, however, that the genetic influences were larger for the decisions made in situations where other group members were making relatively large contributions. This pattern was consistent for the two studies, which employed different procedures; Study 1 was a group experiment and Study 2 was a web experiment.

To see how such genetic and environmental influences on the decisions translated into genetic and environmental influences on game outcomes, we conducted Monte Carlo simulations in Study 3. We found that genetic influences were larger for the outcomes on games with smaller numbers of iterations. As the number of iterations grew, the genetic influences became smaller. However, when the number of iterations increased further, genetic influences recovered. This is because the smaller number of iterations meant that cooperativeness had mostly negative influences on the outcomes because of exploitation by non-cooperators. However, with larger numbers of iterations, cooperativeness could promote repeated cooperation with other cooperators, thus compensating for the loss imposed by non-cooperators. When the negative and positive influences were balanced, individual differences in the outcomes were mostly explained by chance factors (E), making the influences of genetic factors small. However, with a large enough number of iterations, the positive influences of cooperativeness exceeded the negative ones. Thus, individual differences in the outcomes were, again, influenced by the decisions, which were influenced by genetic factors.

The data showed moderate genetic influences on strategies in public goods games. Individual differences in public goods games were shown to be, at least partly, genetically influenced. As natural selection usually produces genetically homogeneous populations in regard to fitness-related traits, the existence of genetic variance poses an enigma (Buss, 1991; Penke et al., 2007; Hiraishi et al., 2008). This is especially so for behavior in social dilemmas because cooperation has played a large role in human evolution (Silk and House, 2011). How have such genetic variances been maintained through natural selection? Our results suggest some possible explanations.

First, the influence of genetic factors was smallest for decisions made in situations where others were not cooperative. This can be explained by selection pressure being strongest in such settings. Our Monte Carlo simulation data in Study 3 showed that being cooperative in such situations has negative influences on the outcomes regardless of the number of game iterations. Genetic factors that made organisms cooperative under less cooperative social settings are more likely to have been selected out through natural selection.

Second, the larger genetic influences in cooperative situations can be explained in the following way. As suggested by the Monte Carlo simulations, being cooperative in cooperative situations can be beneficial as long as the number of game iterations is sufficient. However, free riding is a better strategy when the number of iterations is small. Therefore, the number of iterations determines whether cooperation is a better strategy or not. If the real world provides both short- and long-term relationships, free riding may be a better strategy in the former while cooperation may be more successful in the latter. That is, environmental heterogeneity may have maintained strategy variances that are partly genetic (Penke et al., 2007). Another possibility is that in real world settings, the length of social interaction is at the point where the costs and benefits of adopting a free riding or cooperative strategy are balanced. If this were the case, genetic variances would have been maintained through negative frequency dependent selection (Penke et al., 2007).

Finally, we should be cautious in assuming exact heritability estimates from the current study. We conducted only two experiments with a limited number of twin participants and some of the participants took part in both studies. We should wait for future studies conducted with other samples, ideally from different cultural backgrounds, to decide the exact strength of genetic factors in shaping human cooperativeness. We should also be cautious in evaluating the Study 3 results of the Monte Carlo simulation of virtual iterated games. We used decisions made in Study 2, in which the participants registered their strategies expecting a one-shot game, to simulate their behavior on iterated games. In addition, our “decision makers” in the simulation took into account only the last preceding round. It is possible to think of a simulation where the decision makers have a longer memory; i.e., they compute the average contribution by others in the preceding 10 rounds. Ideally, it would be better to conduct real iterated games with twins to obtain an exact heritability estimation for human cooperativeness in iterated N-person social dilemmas. However, we believe that our data provide some hints with which to understand the genetics and evolution of human prosociality.

## Author Contributions

KH designed the research, analyzed the data, and wrote the paper. CS and JA contributed to conducting the experiments, interpreting the data, and writing the paper. SY contributed to interpreting the data and writing the paper.

## Conflict of Interest Statement

The authors declare that the research was conducted in the absence of any commercial or financial relationships that could be construed as a potential conflict of interest.

## Abbreviations

HC: Highest conditional decision in Study 1
HC2: Highest conditional decision in Study 2
LC: Lowest conditional decision in Study 1
LC2: Lowest conditional decision in Study 2
MC: Medium conditional decision in Study 2
MHC: Middle to high conditional decision in Study 1
MLC: Middle to low conditional decision in Study 1
UC: Unconditional decision in Study 1
UC2: Unconditional decision in Study 2.
